# Supplementary material for: Artificial Intelligence in Predicting Microsatellite Instability and KRAS, BRAF Mutations from Whole-Slide Images in Colorectal Cancer: A Systematic Review
Source: Diagnostics (Basel). 2023 Dec 31;14(1):99. doi: 10.3390/diagnostics14010099 (PMC10795725; doi:10.3390/diagnostics14010099)
Supplement: Supplementary file 1 [file diagnostics-14-00099-s001.zip › Tableau Stage_biomarkers_ttt FIGURE S1.pptx]

## Slide 1
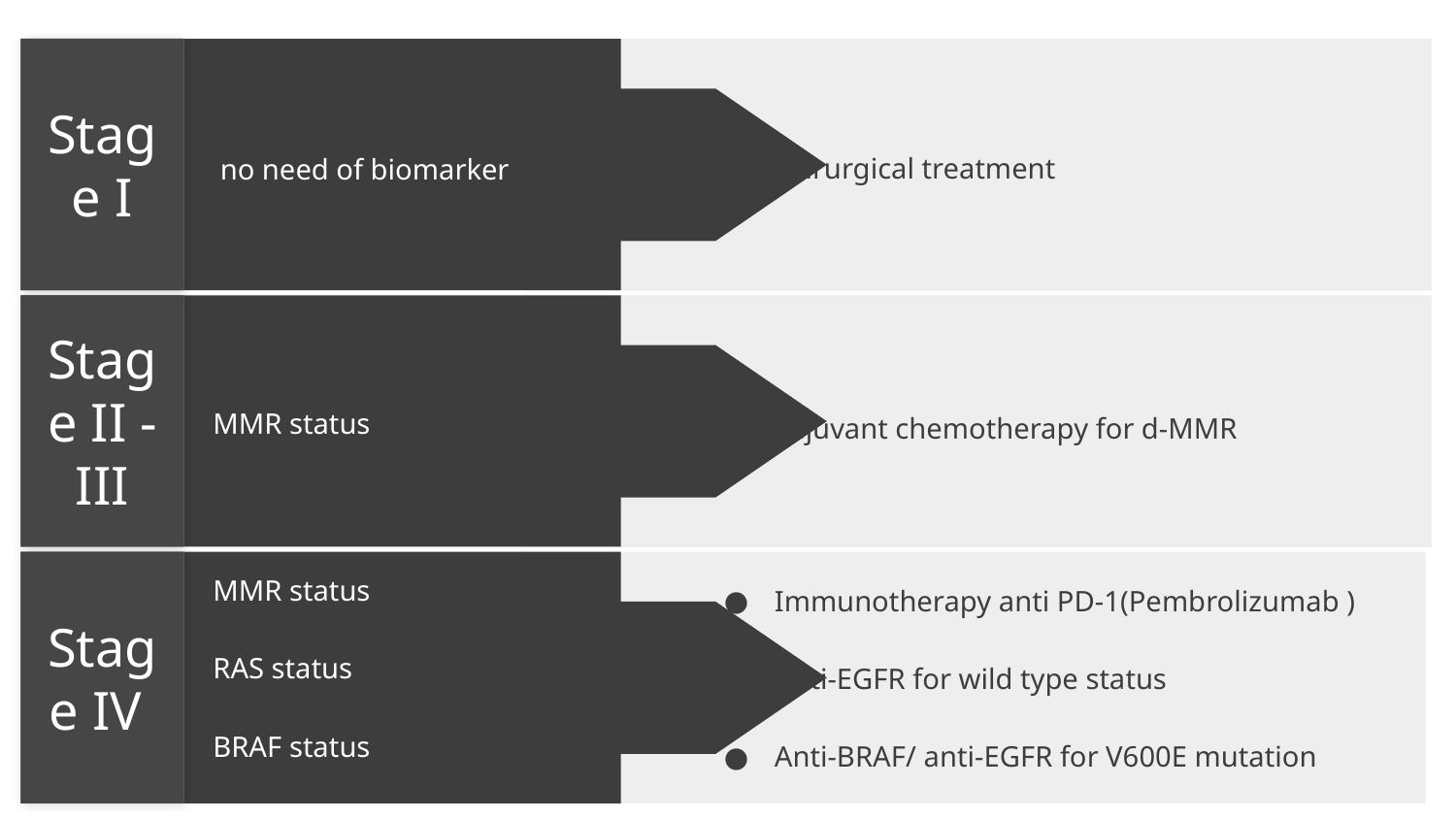

Stage I
Chirurgical treatment
 no need of biomarker
Stage II -III
MMR status
Adjuvant chemotherapy for d-MMR
Immunotherapy anti PD-1(Pembrolizumab )
Anti-EGFR for wild type status
Anti-BRAF/ anti-EGFR for V600E mutation
Stage IV
MMR status
RAS status
BRAF status
