# Supplementary figures and images for: Artificial Intelligence in Predicting Microsatellite Instability and KRAS, BRAF Mutations from Whole-Slide Images in Colorectal Cancer: A Systematic Review
Source: Diagnostics (Basel). 2023 Dec 31;14(1):99. doi: 10.3390/diagnostics14010099 (PMC10795725; doi:10.3390/diagnostics14010099)

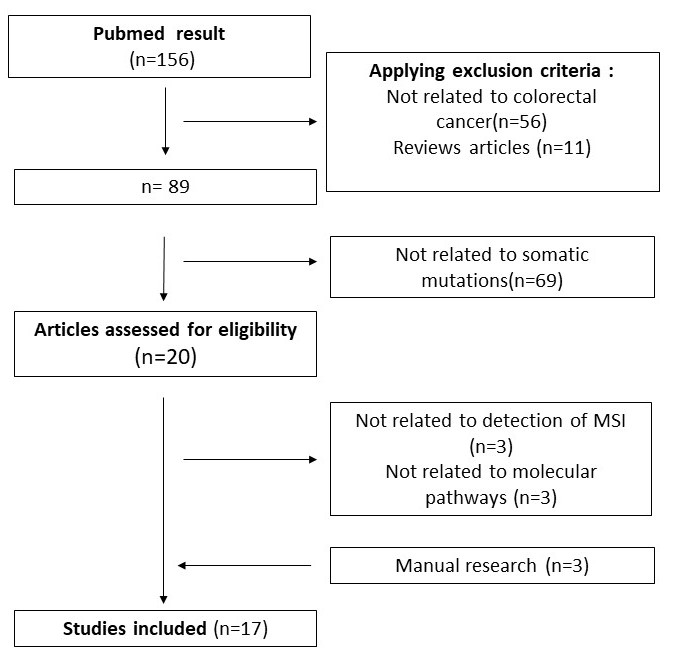

Supplement: Supplementary file 1 [file diagnostics-14-00099-s001.zip › flow chart FIGURE.jpg]
